# Urinary N-Acetyl-beta-D-glucosaminidase as an Early Marker for Acute Kidney Injury in Full-Term Newborns with Neonatal Hyperbilirubinemia

**DOI:** 10.1155/2014/315843

**Published:** 2014-06-24

**Authors:** Bangning Cheng, Yulian Jin, Guanghui Liu, Zhiheng Chen, Hongmei Dai, Min Liu

**Affiliations:** ^1^Clinical Laboratory, Children's Hospital of Anhui Province, Hefei, Anhui 230000, China; ^2^Department of Paediatrics, Children's Hospital of Anhui Province, Hefei, Anhui 230000, China; ^3^Department of Paediatrics, Third Xiangya Hospital of Central South University, Changsha, Hunan 410013, China; ^4^Operation Center, Third Xiangya Hospital of Central South University, Changsha, Hunan 410013, China

## Abstract

*Purpose*. To investigate renal function estimated by markers in full-term newborns with hyperbilirubinemia. *Methods*. A total of 332 full-term newborns with hyperbilirubinemia and 60 healthy full-term newborns were enrolled. Total serum bilirubin, serum creatinine (Cr), serum blood urea nitrogen (BUN), serum cystatin C (Cys-C), urinary beta-2-microglobulin (*β*_2_MG) index, and urinary N-acetyl-beta-D-glucosaminidase (NAG) index were measured before and after treatment. All newborns were divided into three groups according to total serum bilirubin levels: group 1 (221-256), group 2 (256-342), and group 3 (>342). *Results*. The control group and group 1 did not differ significantly in regard to serum Cr, serum BUN, serum Cys-C, urinary *β*_2_MG index, and urinary NAG index. Urinary NAG index in group 2 was significantly higher than that in control group (*P* < 0.001). Between control group and group 3, serum Cys-C, urinary *β*_2_MG index, and urinary NAG index differed significantly. The significant positive correlation between total serum bilirubin and urinary NAG index was found in newborns when total serum bilirubin level was more than 272 *μ*mol/L. *Conclusions*. High unconjugated bilirubin could result in acute kidney injury in full-term newborns. Urinary NAG might be the suitable marker for predicting acute kidney injury in full-term newborns with hyperbilirubinemia.

## 1. Introduction

Neonatal hyperbilirubinemia is defined as a total serum bilirubin level more than 86 *μ*mol/L (5 mg/dL) [1]. Neonatal hyperbilirubinemia resulting in clinical jaundice is one of the most common problems among infants, occurring in up to 60% of healthy full-term newborns and 80% of preterm newborns [2, 3]. Moreover, neonatal hyperbilirubinemia is known to occur more frequently and to be more severe in Asians [4]. Newborns, especially preterm newborns, have higher rates of bilirubin production than adults, because they have red cells with a higher turnover and a shorter life span. Furthermore, deficiency of hepatic uptake, impaired conjugation of bilirubin, and increased enterohepatic circulation of bilirubin could limit the ability to excrete bilirubin and result in hyperbilirubinemia [5, 6].

Most cases of neonatal hyperbilirubinemia are physiological and total serum bilirubin level less than 205 *μ*mol/L (12 mg/dL) usually has no serious consequences [7]. However, the newborn with a total serum bilirubin level more than 342 *μ*mol/L (20 mg/dL) is a concern. The severe hyperbilirubinemia can lead to kernicterus and neurodevelopmental abnormalities such as hearing loss, athetosis, and, rarely, intellectual deficits [8]. The impairment of glomerular filtration and tubular functions had also been observed in newborn with high levels of serum unconjugated bilirubin [9].

Serum creatinine (Cr), serum blood urea nitrogen (BUN), serum cystatin C (Cys-C), urinary beta-2-microglobulin (*β*_2_MG), and urinary N-acetyl-beta-D-glucosaminidase (NAG) were often used to evaluate renal injury [10]. Whether these variables could predict renal injury in full-term newborns with hyperbilirubinemia remains unknown. In this study, we aimed to evaluate renal injury estimated by serum Cr, serum BUN, serum Cys-C, urinary *β*_2_MG, and urinary NAG in full-term newborns with hyperbilirubinemia.

## 2. Materials and Methods

### 2.1. Study Population

All subjects were full-term newborns, with a gestational age of more than 37 weeks, who were born between January 2011 and December 2012. Newborns with antenatal or neonatal asphyxia, temperature abnormality, septicemia, antenatal viral infection, congenital dysmorphia, and congenital heart disease were excluded. We also excluded newborns whose mothers had maternal complications during the pregnancy. Informed consents were obtained from the parents of each newborn. This research protocol was approved by a local research committee.

### 2.2. Determinations of Laboratory Variables

At 48 hours of birth, the venous blood and urine were obtained from newborns without phototherapy or exchange transfusion. Determinations of bilirubin, Cr, and BUN were performed using Cobas 6000 analyzer (Roche, Mannheim, Germany) according to the manufacturer's instructions. Serum total bilirubin was measured using the Bilirubin Total DPD Gen.2 kit (Roche, Mannheim, Germany). Serum and urinary Cr were measured by an enzymatic assay (Creatinine Plus; Roche Diagnostics, Branchburg, NJ). Serum Cys-C was measured by the particle enhanced nephelometric immunoassay (Dade Behring Marburg GmbH, Marburg, Germany). Urinary *β*2-M was measured by radioimmunoassay using Coat A-Count Beta-2 Microglobulin IRMA kit (Diagnostic Products Co., Los Angeles, CA, USA). Urinary NAG was measured using the NAG assay kit (Bio-quant, CA, USA). The ratio of urinary NAG to urinary Cr (NAG index, mg/g Cr) and the ratio of urinary *β*_2_MG to urinary Cr (*β*_2_MG index, U/g Cr) were also determined in spot urine samples.

Phototherapy was used for treatment of newborns with hyperbilirubinemia and exchange transfusion was used for severe neonatal jaundice. Posttreatment studies were done in all newborns until total serum bilirubin levels had decreased to less than 171 *μ*mol/L.

All jaundiced newborns were divided into three groups according to total serum bilirubin levels. Group 1 newborns had total serum bilirubin levels ranging between 221 and 256 *μ*mol/L. Group 2 newborns had total serum bilirubin levels ranging between 256 and 342 *μ*mol/L. Group 3 newborns had total serum bilirubin levels more than 342 *μ*mol/L [11]. Healthy full-term newborns were divided into the control group.

### 2.3. Statistical Analyses

All data were expressed as mean ± standard deviation (SD). Data were compared between different groups by Mann-Whitney *U* test or Kruskal Wallis test as appropriate. Correlations between different markers of renal function were explored by Spearman's rank correlation coefficient. Statistical analysis was performed using SPSS 13.0. All probabilities were two-tailed. Values of *P* < 0.05 were considered as statistically significant.

## 3. Results

A total of 332 newborns with hyperbilirubinemia were enrolled into the study. ABO blood group incompatibility was the most common cause (*n* = 287), followed by glucose-6-phosphate dehydrogenase (G6PD) deficiency (*n* = 45). A total of 60 healthy full-term newborns were used as control. The demographic and clinical data are summarized in Table 1. There were no statistically significant differences in gestational age, birth weight, gender, and delivery between newborns with and newborns without hyperbilirubinemia.

There is no statistically significant difference in urinary pH between different groups. The control group and group 1 did not differ significantly in regard to serum Cr, serum BUN, serum Cys-C, urinary *β*_2_MG index and urinary NAG index (*P* = 0.301, *P* = 0.871, *P* = 0.872, *P* = 0.139, and *P* = 0.432). Serum Cr, serum BUN, serum Cys-C, and urinary *β*_2_MG index in group 2 were higher than those in control group; however, the differences between the two groups did not reach statistical significance (*P* = 0.463, *P* = 0.879, *P* = 0.164, and *P* = 0.132). Urinary NAG index in group 2 was significantly higher than those in control group (*P* < 0.001). Between control group and group 3, serum Cys-C, urinary *β*_2_MG index, and urinary NAG index differed significantly (*P* < 0.001, *P* = 0.014, and *P* < 0.001) (Table 2).

**Figure 1:**
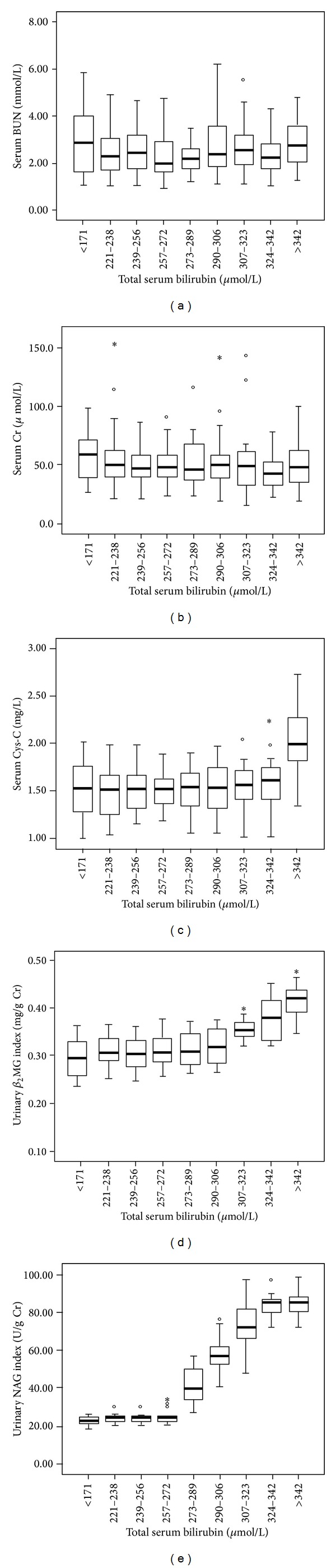
Changes in levels of serum Cr, serum BUN, serum Cys-C, urinary *β*_2_MG index, and urinary NAG index according to different total serum bilirubin levels.

Figure 1 showed the associations between markers and total serum bilirubin. The serum Cr (*r* = −0.041; *P* = 0.455) and BUN (*r* = 0.176; *P* = 0.001) were not related to total serum bilirubin. The significantly increased serum Cys-C level was observed in newborns which had total serum bilirubin levels >342 *μ*mol/L. The levels of urinary *β*_2_MG index significantly increased and were positively correlated with total serum bilirubin levels (*r* = 0.682; *P* < 0.001) in newborns which had total serum bilirubin levels >306 *μ*mol/L. Furthermore, the levels of urinary NAG index significantly increased and were positively correlated with total serum bilirubin levels (*r* = 0.702; *P* < 0.001) in newborns which had total serum bilirubin levels >272 *μ*mol/L.

The time of the posttreatment controls was 32 ± 14 days.  Table 3 showed the markers when total serum bilirubin levels had decreased to less than 171 *μ*mol/L after treatment of hyperbilirubinemia. Between control and newborns with treatment, no statistically significant differences were observed.

## 4. Discussion

Bilirubin, the hydrophobic end product of hemedegradation, is metabolized in the hepatocyte to hydrophilic conjugates, which are then efficiently eliminated in the bile [12]. Traditionally, bilirubin has been regarded as a toxic waste product or the secondary product of physiological conditions. However, it has been recognized as a substance with potent antioxidant and cytoprotective properties through efficient scavenging of peroxyl radicals and suppression of oxidation [13, 14]. High level of bilirubin in the blood leads to hyperbilirubinemia. The bilirubin crystal could result in renal medulla, renal interstitium, and renal tubular necrosis in patients with hyperbilirubinemia.

In this study, we measured serum Cr, serum BUN, serum Cys-C, urinary *β*_2_MG index, and urinary NAG index at 48 hours of birth in 332 full-term newborns with hyperbilirubinemia and 60 healthy full-term newborns. No statistically significant differences of serum Cr and serum BUN levels were observed, even between severe hyperbilirubinemia group (total serum bilirubin >342 *μ*mol/L) and control group. Neonates have a lower muscular mass resulting in lower Cr synthesis. Allegaert et al. demonstrated that Cr assays showed great variation depending on the reaction mechanism and manufacturer. Furthermore, high level bilirubins also disturb the assay of serum and urinary Cr [15, 16]. Consequently, expected Cr values in neonates depended on the specific method used and serum Cr was inappropriate for evaluating renal function in full-term newborns with hyperbilirubinemia.

Because monitoring neonatal urine output is difficult, the application of endogenous creatinine clearance is also limited to monitoring renal function in newborns. The biochemical characteristics of Cys-C allow free filtration in the renal glomerulus and subsequent metabolism and reabsorption by the proximal tubule. Cys-C production in the body is a stable process and is not influenced by renal conditions, increased protein catabolism, or dietetic factors. Moreover, it does not change with muscle mass like Cr does. Therefore, serum Cys-C has been suggested to be an ideal endogenous marker of glomerular filtration rate [17, 18]. *β*_2_MG is a small circulating protein which is partly filtrated at the glomerulus. After glomerular filtration, *β*_2_MG is normally 99.9% reabsorbed in proximal tubule and degraded in the lysosome of the renal tubular cell, and finally it is excreted in the urine [19]. The level of urinary *β*_2_MG is increased in time of impaired renal tubular function. Analysis of isoenzymes of NAG demonstrated that its increased urinary excretion is not dependent on increased filtration across the damaged capillary wall but on increased release by renal tubular cells [20]. The increase of urinary NAG level usually implies that there is renal tubular injury [21]. Therefore, urinary *β*_2_MG and urinary NAG have been considered as markers of renal tubular cell injury.

In our study, we had not found increased serum Cys-C, urinary *β*_2_MG index, and urinary NAG index when total serum bilirubin levels were <272 *μ*mol/L. These findings were similar to previous study [22, 23] and might be explained by the fact that lower unconjugated bilirubin could not result in renal damage of newborns. The significantly increased serum Cys-C was observed in newborns which had total serum bilirubin levels >342 *μ*mol/L. Our data suggested that severe level of unconjugated bilirubin had nephrotoxicity in newborns. We found significantly increased urinary *β*_2_MG index in newborns which had total serum bilirubin >306 *μ*mol/L. We also found that urinary NAG index significantly increased when total serum bilirubin level was >272 *μ*mol/L. Furthermore, positive correlation between total serum bilirubin and urinary *β*_2_MG and positive correlation between total serum bilirubin and urinary NAG index were found in newborns with hyperbilirubinemia. These results demonstrated that the high level of unconjugated bilirubin was directly involved in the impairment of tubular function in newborns with hyperbilirubinemia. Our data indicated that unconjugated bilirubin might result in impairment of renal tubular function firstly. Along with continued increase of total serum bilirubin, damage of glomerular function would also appear.

We measured the levels of markers when total serum bilirubin levels of newborns had decreased to less than 171 *μ*mol/L after treatment of hyperbilirubinemia. No significant differences were seen between hyperbilirubinemia group and control group in any of the measured variables. These results suggested renal injury of hyperbilirubinemia in newborns might be transient and reversible and can be prevented by lowering total serum bilirubin levels to near-normal levels. Our study indicated that high unconjugated bilirubin could result in impairment of renal tubular and glomerular function of full-term newborns. Urinary NAG index might be the suitable marker for predicting acute kidney injury in full-term newborns with hyperbilirubinemia.

Relatively small sample size was a limitation of the present study. Additionally, other markers of renal tubular function, such as urinary lysozyme, urinary *α*1-microglobulin, and urinary CC16, were not studied in this study. The large-scale samples and more markers evaluations are required in further study.

## 5. Conclusions

In conclusion, high unconjugated bilirubin could result in acute kidney injury in full-term newborns. Urinary NAG index might be the suitable marker in predicting acute kidney injury in full-term newborns with hyperbilirubinemia.

## Figures and Tables

**Table 1:** Demographic and clinical data of newborns enrolled in the study.

Data	Hyperbilirubinemia group (*n* = 332)	Control group (*n* = 60)	*P* value
Gestational age (week)	38.6 ± 2.23	38.1 ± 2.02	0.106
Birth weight (g)	3198.1 ± 425.3	3258.5 ± 521.8	0.847
Gender			
Female	198	32	0.361
Male	134	28	
Delivery			
Normal	253	46	0.930
Cesarean section	79	14	

**Table 2:** Distribution of variables levels in different groups.

	Control group (*n* = 60)	Group 1 (*n* = 119)	Group 2 (*n* = 165)	Group 3 (*n* = 48)
Cr (*μ*mol/L)	47.8 ± 18.5	50.9 ± 19.8	49.9 ± 20.3	50.4 ± 18.7
BUN (mmol/L)	2.82 ± 1.32	2.78 ± 1.95	2.85 ± 1.31	2.83 ± 1.16
Cys-C (mg/L)	1.51 ± 0.38	1.52 ± 0.41	1.60 ± 0.54	1.98 ± 0.62
*β*_2_MG (mg/g Cr)	0.29 ± 0.09	0.31 ± 0.07	0.34 ± 0.11	0.38 ± 0.21
NAG index (U/g Cr)	23.1 ± 12.9	24.6 ± 10.2	52.3 ± 20.3	64.4 ± 28.7
Urinary pH	6.4 ± 0.3	6.3 ± 0.2	6.3 ± 0.4	6.3 ± 0.3

**Table 3:** Distribution of variables levels before and after treatment of hyperbilirubinemia.

	Cr (*μ*mol/L)	BUN (mmol/L)	Cys-C (mg/L)	*β*_2_MG (mg/g Cr)	NAG index (U/g Cr)
Before treatment	50.33 ± 21.6	2.53 ± 1.45	1.79 ± 0.78	0.35 ± 0.28	43.6 ± 25.9
After treatment	51.04 ± 35.2	2.67 ± 1.09	1.62 ± 0.51	0.31 ± 0.11	27.6 ± 19.3
Control	47.8 ± 18.5	2.82 ± 1.32	1.51 ± 0.38	0.29 ± 0.09	23.1 ± 12.9
